# An Elucidation of Substrate Effects in Graphene-Based Sensing Characteristics—Interfaces between Organic Solvent and Graphene

**DOI:** 10.1021/acssensors.5c01138

**Published:** 2025-06-17

**Authors:** Yu-Xuan Lu, Guan-Ying Chen, Fang-Min Lin, Ming-Hsiu Tsai, Chih-Ting Lin

**Affiliations:** † Graduate Institute of Electronics Engineering, 33561 National Taiwan University, Taipei 106319, Taiwan; ‡ Graduate Institute of Biomedical Electronics and Bioinformatics, National Taiwan University, Taipei 106319, Taiwan

**Keywords:** suspended graphene, aqueous organic solvents, molecular sensing mechanisms, hysteresis effect, electrostatic force, van der Waals force

## Abstract

Most graphene-sensor researches have focused on direct graphene modifications to enhance performance. However, supporting-substrate effects on graphene sensing mechanisms remain underexplored. Because of graphene 2D architecture, substrates affect its surface potential, wettability, and molecular adsorption. These effects intensify in the presence of polar molecules, e.g., water molecules, further complicating the sensing characteristics. To explore these effects, this study investigates the influence of substrate on the sensing capabilities and mechanisms of graphene field-effect transistors (GFETs) in organic solvents through electrical-transport measurements. Specifically, we compare partially suspended graphene FETs (PS-GFETs) and oxide-supported graphene FETs (OS-GFETs) in response to dimethyl sulfoxide (DMSO), ethanol, and isopropanol (IPA) at different concentrations. By quantifying Dirac-point hysteresis, we experimentally show that the hysteresis correlates with molecular polarity, following the trend DMSO < ethanol < IPA. Moreover, OS-GFETs exhibit a 1.5-fold sensitivity enhancement compared to PS-GFETs when detecting organic solution concentrations. Employing the two-dimensional hydrogen bond network (2D-HBNS) model, we theoretically illustrate that hydrophobic PS-GFET surfaces maintain equilibrium through hydration shell and 2D-HBNS formation. In contrast, hydrophilic OS-GFET surfaces disrupt this balance, enhancing van der Waals interactions and attracting organic molecules. This leads to superior sensitivity in OS-GFETs. To further validate this hypothesis, we introduced poly­(methyl methacrylate) (PMMA) and polytetrafluoroethylene (PTFE) layers on the SiO_2_ substrate. The experiments show it changes graphene-surface hydrophilicity and graphene-sensor sensitivity. These findings establish a theoretical and experimental framework for optimizing graphene-based sensors. This framework elucidates a solute–solvent interfacial interaction model for polar liquids, aiming to improve the sensing characteristics of 2D materials.

## Graphical abstract

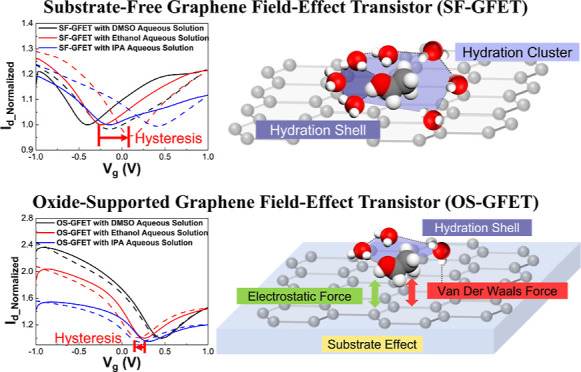

The development of high-performance sensors has become a critical priority in fields ranging from environmental monitoring,^1–4^ biomedical recognition,^5–7^ and industrial manufacturing.^8–10^ Among the wide array of materials explored for sensor applications, graphene has emerged as a highly promising sensing material due to its exceptional intrinsic properties as a two-dimensional (2D) material. These include its high carrier mobility (exceeding 10,000 cm^2^/V·s),^11^ large specific surface area (∼2600 m^2^/g),^12^ and excellent chemical stability,^13^ making graphene well-suited for sensitive detection.^14,15^ Significant progresses have been made in enhancing the sensitivity and selectivity of graphene-based sensors.^16–19^ Among various strategies, surface modification techniques—such as functionalized graphene, graphene oxide, and reduced graphene oxide—have been widely employed to enhance interaction with target molecules and improve sensitivity.^20–22^ However, these modifications often increase structural heterogeneity and reduce electrical conductivity, detrimentally affecting sensor performance.^23,24^ As an alternative to direct graphene modification, recent research has increasingly focused on the use of supporting substrates to modulate the properties of graphene, offering a promising strategy to maintain or enhance the performance without compromising structural integrity.

Prior studies have demonstrated that the supporting substrate plays a pivotal role in modulating the physical and chemical properties of graphene-based sensors. Physically, the substrate influences key electronic parameters, such as doping levels and carrier mobility, primarily through interactions between the substrate and the π-electron system of graphene. For example, hydrophilic substrates, such as oxygen-plasma-treated SiO_2_, are known to enhance p-type doping.^25^ Chemically, the substrate affects the reactivity and surface functionalization of graphene by modulation of its interfacial chemical environment. This is exemplified by the formation of graphene through the hydrogenation of graphene on nickel (Ni) substrates, in contrast to the chemically inert behavior observed with substrates such as platinum (Pt) and copper (Cu), where molecular adsorption is significantly suppressed.^26,27^ Despite these insights, limited research has systematically investigated the underlying sensing mechanisms and performance differences between suspended and substrate-supported graphene configurations, particularly in the detection of complex polar molecules. In our previous studies, we demonstrated that water molecules at the graphene interface arrange themselves into a two-dimensional hydrogen-bond network structure (2D-HBNS).^28^ The 2D-HBNS can be altered by introducing ions and enabling ion concentration detection.^29^ However, when a supporting substrate is presented, the surface properties of graphene are further altered, impacting charge transfer, adsorption dynamics, and molecular recognition.^30,31^ Uncovering these substrate-influence sensing behaviors of graphene is essential for elucidating the interfacial charge dynamics and optimizing the detection performance.

To investigate the influence of substrates on the sensing behavior of graphene, this study compares the performance of partially suspended graphene field-effect transistors (PS-GFETs) and oxide-supported GFETs (OS-GFETs) in the detection of polar organic molecules. Organic solvents were selected instead of organic gases to better elucidate surface interactions, as solid–liquid interfaces promote denser molecular accumulation and more structured interfacial environments compared with the disordered and weakly oriented molecular layers typically formed in gas-phase adsorption. In this work, three solvents, dimethyl sulfoxide (DMSO), ethanol, and isopropyl alcohol (IPA), with different polarities and molecular weights were tested at various concentrations. Dirac-point hysteresis measurements were employed to probe the molecular interactions at the graphene surface. Experimentally, PS-GFETs and OS-GFETs exhibited varying degrees of Dirac-point hysteresis depending on the type and concentration of the organic solvent. By correlating the measurement results with the 2D-HBNS model in the presence of organic molecules, we established the interfacial molecular mechanism associated with the supporting substrate during the gate voltage sweeps. To further validate the proposed mechanism, surface wetting experiments were performed by introducing PMMA and PTFE interlayers between the SiO_2_ substrate and graphene. These findings offer fundamental insights into the interfacial processes governing graphene sensor behavior and provide guidance for the optimization of graphene-based sensors for organic molecule detection in chemical, pharmaceutical, and environmental applications.

## Device and Method

### Graphene Fabrication and Transfer

In our experiments, double-layer graphene was selected for its enhanced electronic properties and mechanical strength. Initially, monolayer graphene films were synthesized by using chemical vapor deposition (CVD) in a high-temperature furnace. During the fabrication process, we used copper foil as the catalytic substrate and methane as the carbon source.^32^ The synthesized graphene films were then detached and physically stacked to form double-layer graphene with the support of PMMA through the electrochemical-bubble delamination transfer method.^33^ Deionized water was utilized throughout all experimental procedures to ensure a clean and controlled environment.

### GFET Structure and Fabrication Process

Partially suspended graphene field-effect transistor (PS-GFET) was fabricated using suspended graphene positioned over a micropost array with two microreservoirs and electrodes, as illustrated in Figure 1a. The micropost array and microreservoir patterns were defined using photolithography on a silicon wafer with thermally grown silicon dioxide (SiO_2_), followed by reactive ion etching (RIE) to remove 300 nm of SiO_2_ and 30 μm of silicon in the patterned areas. The resulting pillar array consisted of a SiO_2_/Si composite structure, comprising 5 μm × 5 μm square SiO_2_/Si pillars spaced at a 5 μm pitch. Source and drain electrodes were deposited via an E-Gun evaporator, consisting of a 5 nm titanium adhesion layer followed by a 60 nm gold layer. The physically stacked bilayer graphene, supported by PMMA, was then transferred over the etched trench. To complete the fabrication, the devices were immersed in acetone for 24 h to dissolve the PMMA layer, followed by rinsing with isopropanol (IPA) and deionized water to remove residual contaminants. The completed PS-GFET device had a total area of 2 × 2 cm.

**Figure 1:**
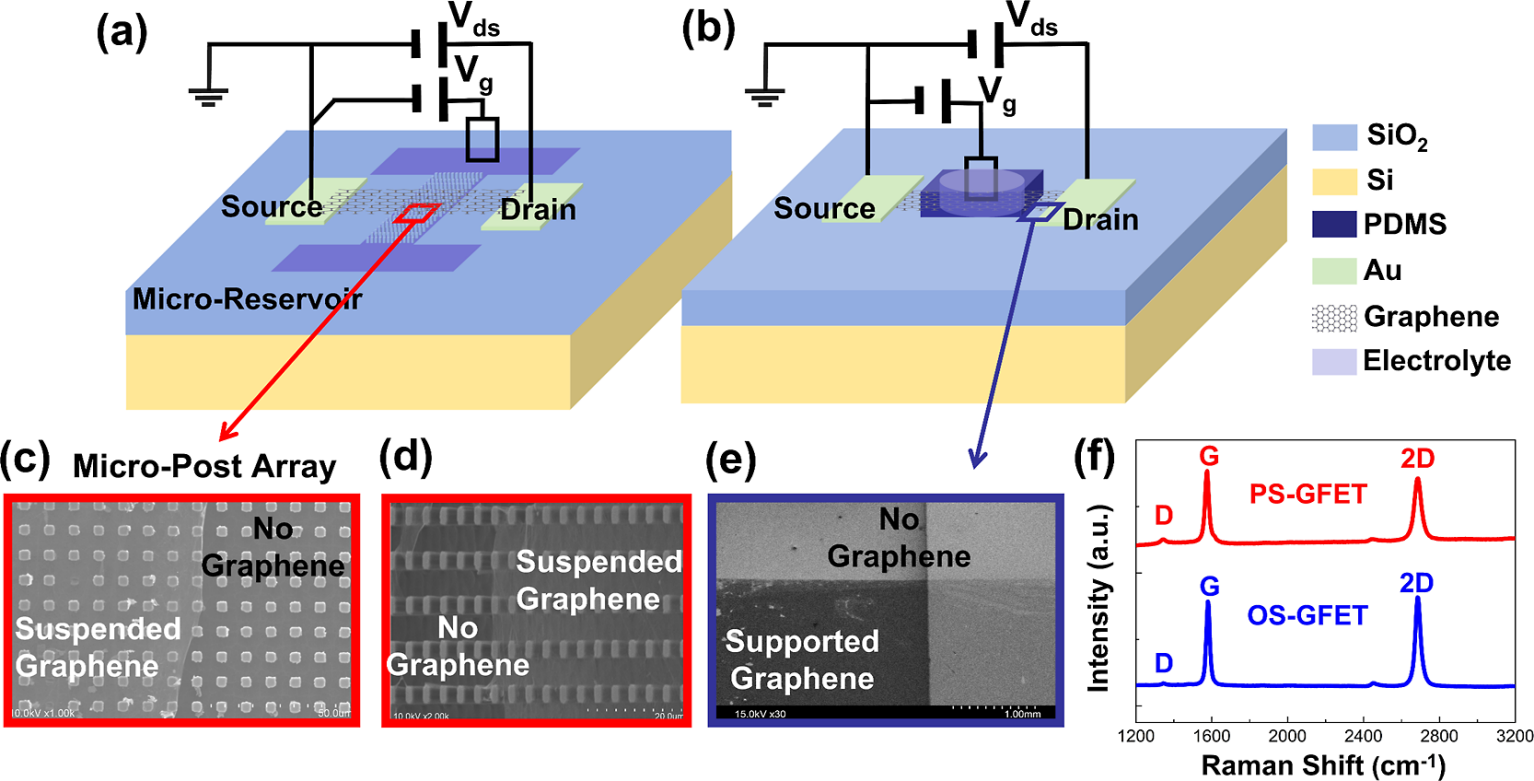
Schematic view of the PS-GFET/OS-GFET device and measurement diagram: (a) PS-GFET device; (b) OS-GFET device; (c) SEM image of graphene partially suspended on micropost array in the PS-GFET device; (d) tilted-view SEM image of the PS-GFET taken at a 60° angle from the horizontal; (e) SEM image of supported graphene on substrate in the OS-GFET device; (f) Raman spectroscopy of partially suspended graphene on micropost array in the PS-GFET device (red line) and supported graphene on the OS-GFET device (blue line).

In contrast, an oxide-supported graphene field-effect transistor (OS-GFET) was fabricated with bilayer graphene directly placed on an SiO_2_ substrate, with two electrodes positioned on either side, as depicted in Figure 1b. The OS-GFET fabrication process direct deposited the electrodes on a SiO_2_/Si substrate without the need for microchannel design. After transferring the stacked bilayer graphene and removing the PMMA support, a polydimethylsiloxane (PDMS) chamber was put on top of graphene to serve as the gate electrolyte reservoir.

For graphene wetting experiments, two additional oxide-substrate-supported graphene field-effect transistors were fabricated: the oxide-PMMA-supported graphene field-effect transistor (OPMS-GFET) and the oxide-PTFE-supported graphene field-effect transistor (OPTS-GFET). In the fabrication of OPMS-GFET, a PMMA layer was spin-coated onto graphene as a support layer. Then, the graphene/PMMA stack was transferred onto the SiO_2_ substrate. This step places the PMMA between SiO_2_ and graphene by flipping the stack. Electrode deposition was subsequently performed using a shadow mask. For the OPTS-GFET, a polytetrafluoroethylene (PTFE) layer was spin-coated onto the SiO_2_ substrate before the graphene layer. The electrodes were then deposited on the substrate, completing the device fabrication.

### GFET Characterization

For quality control, electron microscopy (SEM) and Raman spectroscopy were performed on the fabricated GFETs to validate the quality of the graphene. Figure 1c,d presents top-view and 60°-tilted SEM images of the PS-GFET microfluidic channel. In these figures, a clear contrast between the graphene-covered and graphene-free regions is observed. It confirms the successful suspension of large-area graphene. The tilted view further reveals the morphology of the suspended graphene spanning across the micropost array. Similarly, the SEM image of the OS-GFET confirms the successful fabrication of large-area supported graphene, as shown in Figure 1e. Raman spectroscopy results, displayed in Figure 1f, further confirm the presence of graphene in both PS-GFET and OS-GFET. For the PS-GFET, the G band appears at 1580 cm^–1^, while for the OS-GFET, it is located at 1575 cm^–1^. The shift of the G band in the PS-GFET is attributed to reduced electrostatic doping and enhanced tensile strain in the suspended structure, consistent with previous reported characteristics of suspended graphene.^19,34^ The *I*_D_/*I*_G_ ratio is 0.04 for the PS-GFET and 0.03 for the OS-GFET. These low *I*_D_/*I*_G_ ratios indicate the production of low-defect graphene. Additionally, the *I*_2D_/*I*_G_ ratio is 0.9 for the PS-GFET and 1.05 for the OS-GFET. These values confirm the successful fabrication of both partially suspended and supported bilayer graphene structures.^35,36^

Regarding the electronic properties, carrier mobility was measured to assess the extent of interfacial scattering induced by the SiO_2_ substrates in PS-GFET and OS-GFET devices. The measured mobilities for OS-GFETs and PS-GFETs are approximately 1000 cm^2^/V·s and 1300 cm^2^/V·s, respectively, indicating comparable performance. This modest difference suggests that within our experimental scope, the effect of partial or full substrate support on the sensing performance due to mobility variation is limited.

### Measurement Diagram

The electrical characteristics of all devices were measured by using a Keithley B1500A Semiconductor Characterization System. We recorded and analyzed the electrical transport behaviors by applying a gate voltage sweep from −1 V to +1 V and then from +1 V to −1 V with a fixed source-drain voltage (*V*_sd_) of 0.1 V. The ±1 V sweep range was selected to avoid electrochemical reactions near the water electrolysis threshold (1.23 V) and to ensure stable measurement conditions. A detailed justification for this voltage range selection is provided in the Supporting Information (Electrical Measurement Range Selection). The scanning step rate was set to 20 mV/step.

The gate voltage was applied through the electrolytes. Specifically, in PS-GFETs, gate voltage was applied to electrolytes introduced through the microreservoirs positioned at both ends of the microfluidic channel (Figure 1a), ensuring precise voltage delivery to the graphene–electrolyte interface. All experiments were performed on at least three independently fabricated PS-GFET and OS-GFET devices, with each electrical measurement repeated three times to ensure reproducibility.

Voltage applied in this way can be precisely delivered to the graphene–electrolyte interface. For substrate-supported graphene-FETs, including OS-GFETs, OPMS-GFETs, and OPTS-GFETs, the gate voltage was applied through electrolytes contained in a PDMS reservoir (Figure 1b). Aqueous organic solvents, dimethyl sulfoxide (DMSO), ethanol, and isopropyl alcohol (IPA), at volume concentrations of 10%, 30%, 50%, 70%, and 90% worked as top-gate electrolytes in these experiments.

### Dirac-Point Hysteresis Value and Hysteresis Window

We utilize the Dirac-point hysteresis value and the hysteresis window to quantify changes at the graphene–substrate interface. Graphene band structure is highly susceptible to modulation by external factors.^37^ External influences, such as applied voltage and the accumulation of surface polar molecules or ions, significantly affect the electronic states.^38,39^ These affected states can be observed by the shift, i.e., hysteresis, in the charge neutrality point (Dirac point) under sweeping gate voltages. Therefore, Dirac-point hysteresis can promptly reflect interfacial changes at the graphene surface and make it a reliable sensing indicator.

In our experiments, the Dirac-point hysteresis value (Δ*V*) is defined by the difference between the charge neutrality points during the forward (*V*_FCNP_, −1 V to +1 V) and backward (*V*_BCNP_, +1 V to −1 V) sweeps, expressed as^40^

1 $$\Delta V=V_{BCNP}-V_{FCNP}$$

Simultaneously, we define the hysteresis window (HW) as the absolute value of the hysteresis

2 HW=|ΔV|

The sign of Δ*V* indicates charge dissipation or accumulation on the surface, while the magnitude of HW is proportional to the extent of surface interactions.

## Results and Discussion

### Measurement of Organic Solvents at a Fixed Concentration Using PS-GFET and OS-GFET

This experiment investigates the differences in Δ*V* and HW measured using PS-GFET and OS-GFET for 10% *v*/*v* DMSO, ethanol, and IPA. Through this comparison, we can determine the influence of the supporting substrate and assess the effects of organic molecules with different molecular weights and polarities on the measurements.

Both the PS-GFET and OS-GFET Dirac-point hysteresis phenomena, measured separately in electrolytic solutions containing 10% (*v*/*v*) DMSO (black), ethanol (red), and IPA (blue), are presented in Figure 2a,b. In these figures, the normalized drain current (*I*_d_Normalized_) represents the drain current values normalized by the forward Dirac-point current to eliminate device-to-device variations. The solid line denotes the normalized *I*_d_ values during the forward gate voltage sweep from −1 V to +1 V, while the dotted line represents the values during the backward sweep from +1 V to −1 V.

**Figure 2:**
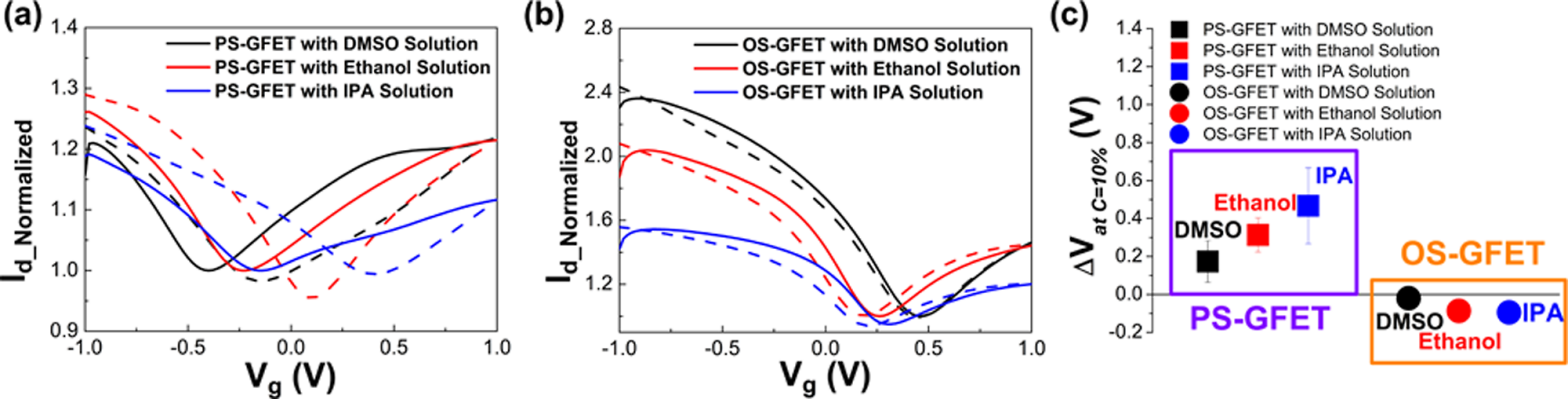
Comparison of graphene transport behavior of PS-GFET and OS-GFET in 10% (*v*/*v*) DMSO aqueous solution electrolyte, 10% (*v*/*v*) ethanol aqueous solution electrolyte, and 10% (*v*/*v*) IPA aqueous solution electrolyte (solid line: transport behavior in the forward scan; dotted line: transport behavior in the backward scan): (a) *I*_d_Normalized_–*V*_g_ curve of PS-GFET; (b) *I*_d_Normalized_–*V*_g_ curve of OS-GFET; and (c) experimental hysteresis effect (Δ*V*) of PS-GFET (purple square) and OS-GFET (orange square).

To facilitate visual comparison, we further calculated the Δ*V* values, as shown in Figure 2c. By comparing the Δ*V* values, we first observed that for the PS-GFET, the Δ*V* values for all three solutions were positive, whereas for the OS-GFET, the hysteresis values were negative. Positive hysteresis indicates that interfacial molecules donate electrons, while negative hysteresis suggests that interfacial molecules withdraw electrons. These differences in hysteresis behavior highlight the significant impact of the supporting oxide layer at the graphene–electrolyte interface. Furthermore, for the PS-GFETs, the statistical hysteresis values were 0.17 ± 0.11 V for DMSO, 0.31 ± 0.09 V for ethanol, and 0.47 ± 0.20 V for IPA. In comparison, for the OS-GFETs, the hysteresis values were-0.02 ± 0.04 V for DMSO, −0.09 ± 0.01 V for ethanol, and −0.10 ± 0.03 V for IPA. It can be concluded that the hysteresis window (HW) of both PS-GFET and OS-GFET followed the behavior: DMSO < ethanol < IPA. The results indicate that the magnitude of HW is inversely proportional to molecular polarity, with no obvious correlation to molecular weight.

Based on the analysis, the supporting oxide substrate significantly influences the graphene surface properties. This leads to distinct electrical performance differences between PS-GFETs and OS-GFETs. This also suggests that graphene experiences different behaviors depending on whether it is partially or fully supported by SiO_2_. Furthermore, the variation in hysteresis behavior among different organic solvents suggests its potential application in solution sensing.

### Measurement of Organic Solvents with Progressively Increasing Concentrations Using PS-GFET and OS-GFET

In this experiment, we measured DMSO, ethanol, and IPA at volume concentrations of 10%, 30%, 50%, 70%, and 90% using PS-GFETs and OS-GFETs. Through these measurements, we can assess the sensitivity of both types of GFETs to changes in organic solvent concentration and further analyze the impact of different solvents on the sensitivity. Figure 3a–c present the Dirac-point hysteresis current curves of PS-GFETs with DMSO, ethanol, and IPA at the specified volume concentrations, while Figure 3d–f illustrate the corresponding curves for OS-GFETs. Consistent with previous results, PS-GFETs exhibit positive hysteresis values across all electrolyte concentrations, whereas OS-GFETs demonstrate negative hysteresis effects for all electrolytes. This phenomenon further emphasizes the influence of the supporting oxide substrate in modulating the hysteresis behavior of OS-GFETs.

**Figure 3:**
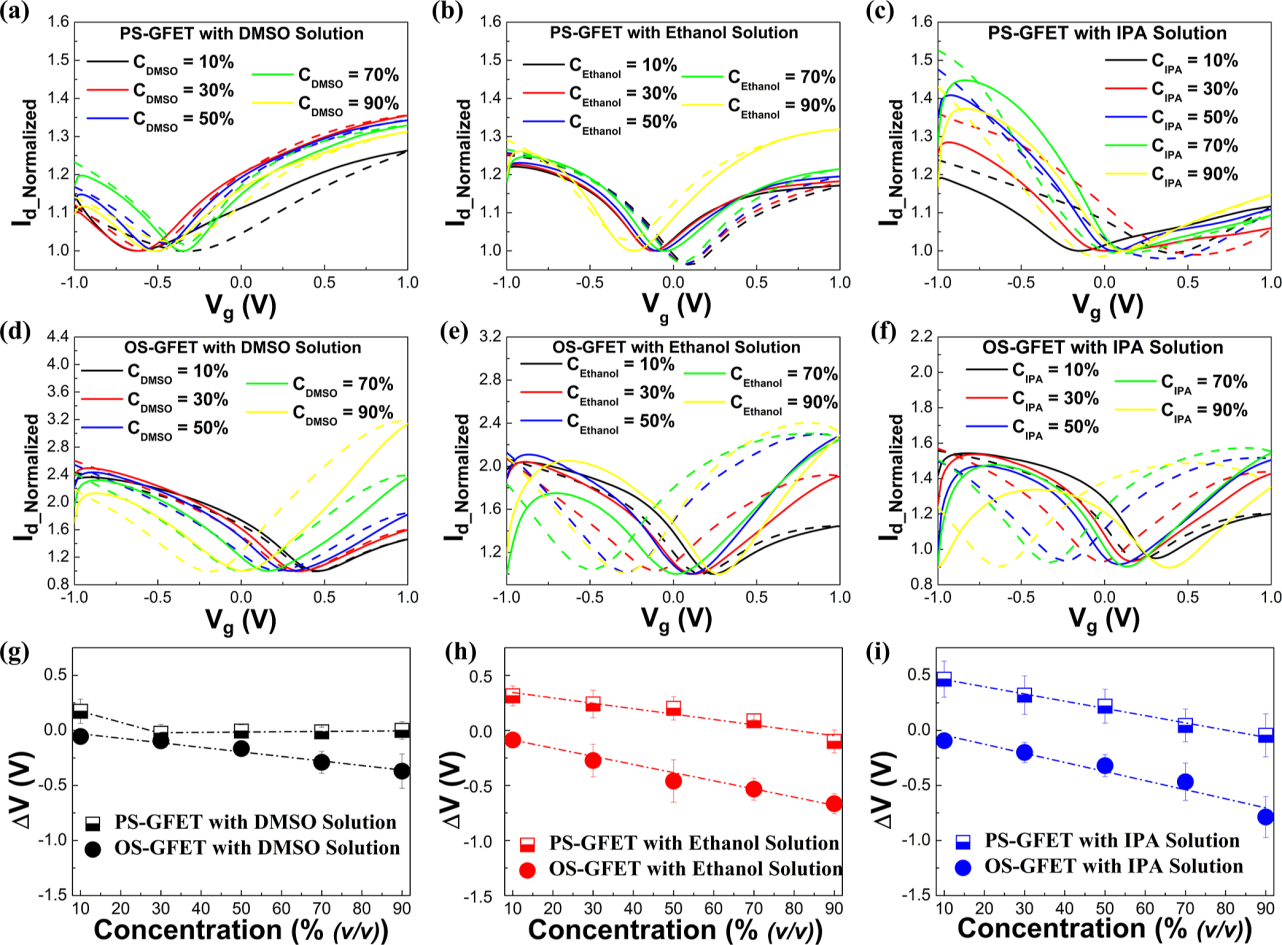
Comparison of transport behavior between PS-GFET and OS-GFET in electrolyte with the concentration ranging from 10% (*v*/*v*) to 90% (*v*/*v*). Solid Line: Transport behavior in forward scan; dotted line: Transport behavior in backward scan. *I*_d_Normalized_–*V*_g_ curve of PS-GFET in different electrolytes: (a) DMSO; (b) ethanol; and (c) IPA. *I*_d_Normalized_–*V*_g_ curve of OS-GFET in different electrolytes: (d) DMSO; (e) ethanol; and (f) IPA. Hysteresis effect (Δ*V*) of PS-GFET and OS-GFET (dotted line represents fitting line): (g) DMSO; (h) ethanol; and (i) IPA.

To clearly visualize the impact of concentration changes, Figure 3g–i illustrate the Δ*V* for PS-GFETs and OS-GFETs in DMSO, ethanol, and IPA solutions. For both SF-FETs and PS-GFETs, Δ*V* gradually decreases with an increasing concentration. It follows an approximately linear relationship, as indicated by the dashed-line fitting results. Notably, in DMSO solutions, the Δ*V* of PS-GFETs declines sharply between 10% and 30% concentration before stabilizing near zero. These results demonstrate that both PS-GFETs and OS-GFETs exhibit good sensing linearity and hold promise as potential sensors for organic solvent detection.

To compare the performance of PS-GFET and OS-GFET in different solutions, we introduce a sensitivity parameter to quantify how the hysteresis varies with changes in solution concentration. The sensitivity is defined as

3 $$sensitivity=\frac{\Delta \left(HW\right)}{\Delta C}$$

In this equation, Δ­(HW) represents the change in the hysteresis window; Δ*C* denotes the change in the solution concentration. The concentration change is derived from the portion of Δ*V* that shows an approximately linear relationship with the concentration.

Figure 4a illustrates the sensitivities of PS-GFETs and OS-GFETs in different solutions. For PS-GFETs, the sensitivity values obtained in 10%–30% DMSO, 30%–90% DMSO, ethanol, and IPA aqueous solutions are 0.01 ± 0.0026 V/[% (*v*/*v*)], 0.0004 ± 0.0002 V/[% (*v*/*v*)], 0.0049 ± 0.0024 V/[% (*v*/*v*)], and 0.0054 ± 0.0023 V/[% (*v*/*v*)], respectively. In comparison, the sensitivity values for OS-GFETs in DMSO, ethanol, and IPA solutions are 0.0041 ± 0.0015 V/[% (*v*/*v*)], 0.0071 ± 0.0006 V/[% (*v*/*v*)], and 0.0083 ± 0.0019 V/[% (*v*/*v*)], respectively. By comparing the sensitivity of both GFETs in different solutions, it can be observed that OS-GFETs exhibit approximately 1.5 times higher sensitivity than PS-GFETs do in ethanol and IPA. In addition, OS-GFETs have 10 times higher sensitivity than PS-GFETs do in 30%–90% DMSO. The early saturation of PS-GFETs in DMSO indicates that they maintain linear behavior only within a narrow concentration range, whereas OS-GFETs exhibit a broader linear response. Additionally, the smaller error bars in OS-GFET sensitivity indicate greater stability, suggesting that the supporting substrate enhances the sensing consistency and reduces the variability.

**Figure 4:**
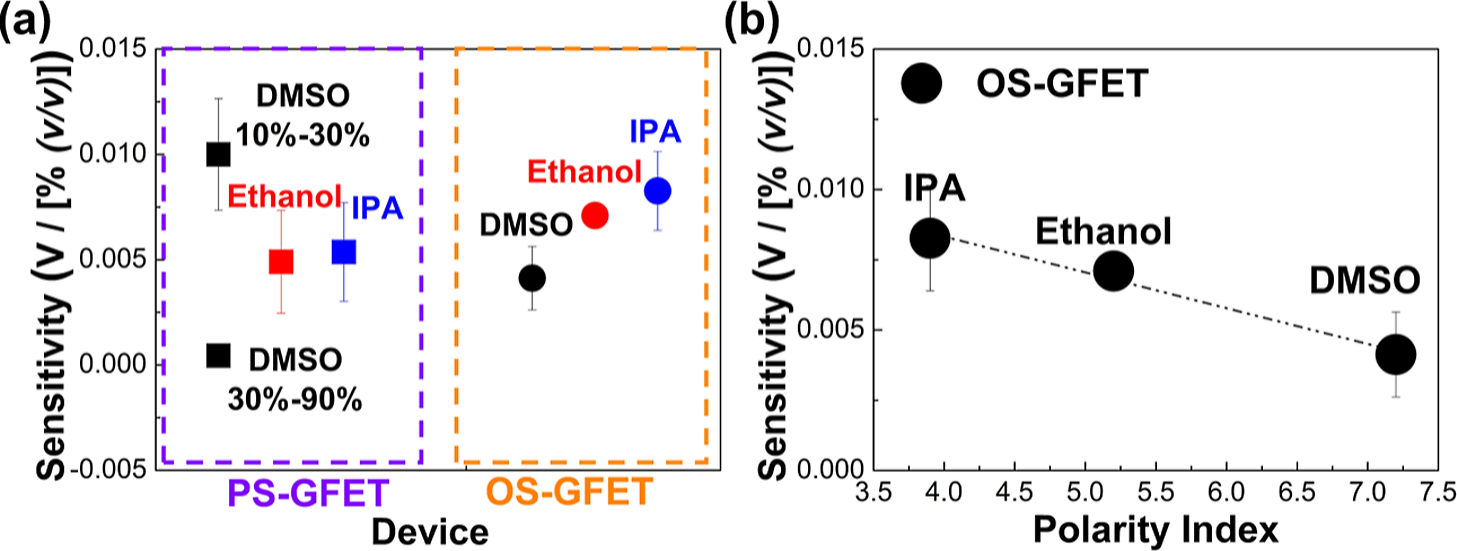
(a) Sensitivity of PS-GFET and OS-GFET in different solutions (IPA, ethanol, and DMSO). (b) Sensitivity of OS-GFET with the corresponding solvent polarity index (the dotted line represents the fitting line).

Figure 4b further demonstrates the relationship between OS-GFET sensitivity and solvent polarity. The results indicate that OS-GFET sensitivity decreases as the polarity index increases, following a near-linear behavior. This suggests that higher solvent polarity weakens graphene–solvent interactions, leading to reduced sensitivity.

Overall, these observations demonstrate that the supporting substrate plays a crucial role in improving both the sensing capability and the stability of OS-GFETs. This improvement is attributed to changes in interfacial interactions between OS-GFETs and organic molecules, which are strongly correlated with the solvent polarity, ultimately leading to distinct sensing characteristics.

### Equilibrium of 2D-HBNS and Hydration Energy Sensing in Partially Suspended Graphene

To elucidate the surface chemical interactions of partially suspended graphene, we simplify the analysis by introducing the concept of 2D-HBNS in a pure water solution.^41,42^ The hydrophobic characteristics^43^ of suspended graphene facilitates the dissociation of water molecules into hydroxide (OH^–^) and hydrogen (H^+^) ions.^44–46^ As shown in Figure 5a, OH^–^ ions tend to accumulate in the first layer of water near the graphene–water interface (first water layer), while H^+^ ions are more likely to reside in the second layer of water. This ion distribution contributes to the formation of the 2D-HBNS. During voltage sweeping, the reversible transition between free-state water molecules (dangling water molecules) and the 2D-HBNS structure induces hysteresis in graphene’s electrical performance.^47,48^ The detailed explanations are provided in the Supporting Information (Water Molecule Configuration at Suspended Graphene Interface).

**Figure 5:**
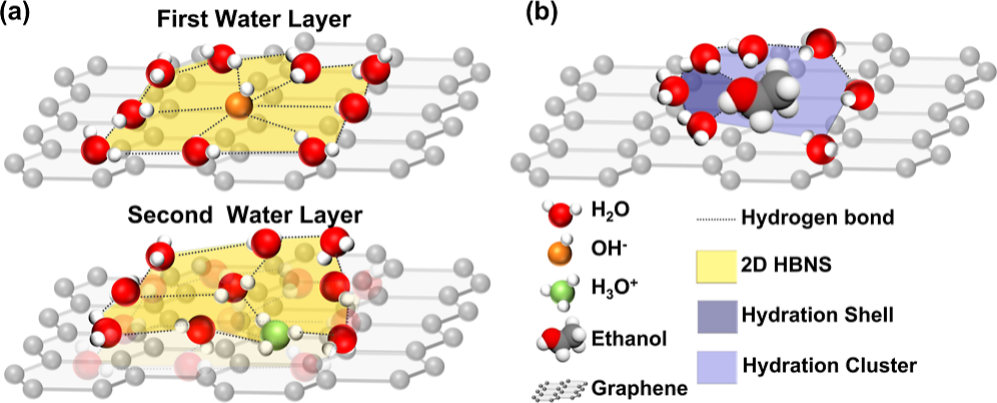
Schematic interfacial diagram of PS-GFET with 10% (*v*/*v*) ethanol aqueous solution: (a) state of water molecules at the graphene–electrolyte interface. It contains the two-dimensional hydrogen bond network structure of water at the first water layer (top figure) and the second water layer (bottom figure); (b) state of ethanol molecules at the graphene–electrolyte interface. Around the ethanol molecule, water molecules tightly bind with the polar portions of the ethanol. These water molecules form the strong hydration shell around the ethanol molecule.

In the presence of organic molecules, two primary mechanisms, hydration interactions with water and van der Waals interactions with the graphene surface, govern the behavior of organic molecules at the graphene interface. Figure 5b illustrates this using ethanol as an example. In the first mechanism, organic molecules tend to attract water molecules^49,50^ due to their polar functional groups, such as hydroxyl or sulfinyl groups, which facilitate hydrogen bonding with interfacial water molecules. This interaction leads to the formation of hydration shells, where water molecules tightly surround the organic molecules, stabilizing them in the aqueous environment.^50,51^ In the second mechanism, the interaction between graphene and organic molecules is primarily governed by van der Waals forces, particularly as the molecules approach the surface. Since graphene lacks polar functional groups, its direct interaction with the polar regions of organic molecules is limited. Instead, the nonpolar regions play a dominant role in adsorption, as dispersion forces (van der Waals interactions) strongly attract the nonpolar region to the graphene surface.^52^ We summarize the changes in interfacial carrier induced by organic molecules in the 2D-HBNS model as

4 $$Q_{\left(\Delta V\right)}=Q_{\left(Hyd\right)}-Q_{\left(VdWF\right)}-Q_{\left(HS\right)}$$

where *Q*_(Δ*V*)_ represents the accumulated charge in the graphene channel due to interfacial interaction. Specifically, *Q*_(Hyd)_ generated through hydrolysis driven by surface hydrophobic interactions contributes to charge accumulation. *Q*_(VdWF)_ arising from the charge polarization of organic molecules adsorbed via van der Waals forces induces negative hysteresis. In contrast, *Q*_(HS)_ reflects the charge variation induced by the shielding effect of the hydration shell, which reduces the charge accumulation. The signs of these charge components correspond to the accumulation of positive or negative charges at the graphene interface. The combined effects of these mechanisms are influenced by organic solvent polarity and concentration.

We have demonstrated that the hysteresis value of PS-GFETs is inversely proportional to the polarity of organic solvents at 10% (*v*/*v*) (Figure 2). From an interfacial molecular perspective, DMSO, with its sulfinyl group (–S=O), exhibits a higher polarity and greater electronegativity than the hydroxyl groups (−OH) in both IPA and ethanol. Its strong polarity stabilizes hydration clusters (*Q*_(HS)_ increase), reduces dangling water molecules (*Q*_(Hyd)_ decrease), and results in a lower hysteresis value (*Q*_(Δ*V*)_ decreases). In contrast, although IPA and ethanol also form hydration clusters, the water molecules within these clusters are more loosely bound, resulting in higher hysteresis values. These observations are consistent with simulations of organic hydration clusters.^53^ The difference in the hysteresis between IPA and ethanol arises from the hydrophobic effects of their nonpolar groups. Compared with ethanol, the branched structure of IPA weakens hydration clusters and increases the number of dangling water molecules at the interface, leading to a larger hysteresis value for IPA than for ethanol. Consequently, the Δ*V* follows the trend: DMSO < ethanol < IPA.

Through experiments measuring organic solvents at varying concentrations (Figure 3), the hysteresis values of PS-GFETs in IPA and ethanol show an approximately linear dependence on concentration, whereas in DMSO, the hysteresis reaches the measurement limit within a narrow concentration range. As previously discussed, the high polarity of DMSO accelerates the formation of the hydration shell (*Q*_(HS)_ increases and *Q*_(Hyd)_ decreases). The strong hydration shell aggregates with near-surface waters, eliminating dangling water molecules and shielding surface charge changes induced by concentration variations, leading to early hysteresis saturation. Meanwhile, *Q*_(VdWF)_ remains nearly constant due to distance constraints. In contrast, the nonpolar chains of IPA and ethanol reduce the hydration shell clustering and interact more readily with graphene. As a result, *Q*_(HS)_ increases and *Q*_(Hyd)_ decreases due to enhanced hydration with rising concentration, while *Q*_(VdWF)_ also increases. These combined effects contribute to the observed linear hysteresis trend. However, despite the differences in polarity between IPA and ethanol, we observed that PS-GFET sensitivity exhibits no significant difference (Figure 4). This is attributed to the limited contribution of *Q*_(VdWF)_ due to distance constraints and the pronounced interference of hydration interactions *Q*_(Hyd)_ with the graphene surface, preventing a clear distinction between the two solvents.

In brief, partially suspended graphene-based sensors for organic molecule detection are constrained by hydrolysis and hydration effects, which predominantly affect distance-dependent interactions. These factors collectively contribute to hysteresis and limits sensitivity.

### Interactions-Driven Sensing in SiO_2_-Supported Graphene

The surface of graphene supported by SiO_2_ is hydrophilic,^54,55^ which results in significant differences in behavior compared to suspended graphene. In a pure water solution, the hydrophilic graphene/SiO_2_ surface has dangling bonds that form silanol groups (Si–OH). As illustrated in Figure 6a, these silanol groups induce the orientation of water molecules toward the interface via electrostatic force,^56^ leading to collective orientation behavior.^57^ This interaction disrupts 2D-HBNS and reduces the number of dangling water molecules. Unlike partially suspended graphene, when organic molecules are introduced into the solution, electrostatic attractions from the silanol groups at the graphene/SiO_2_ interface dominate surface interactions. This attraction reduces the distance between organic molecules and the interface. As the organic molecules approach the graphene surface, van der Waals forces between the molecules and graphene become more pronounced (Figure 6b). This proximity facilitates the formation of a dipole-aligned interfacial layer composed of water and organic molecules as an electric double layer. The formation of this interfacial structure generates an electrostatic gating effect that modulates the Fermi level of graphene. Furthermore, the interfacial hydration shells of organic molecules weaken due to the influence of silanol groups on the substrate. During the backward sweep process, the oriented organic molecules are more likely to remain aligned with the surface, leading to an organic molecule-induced negative hysteresis effect.

**Figure 6:**
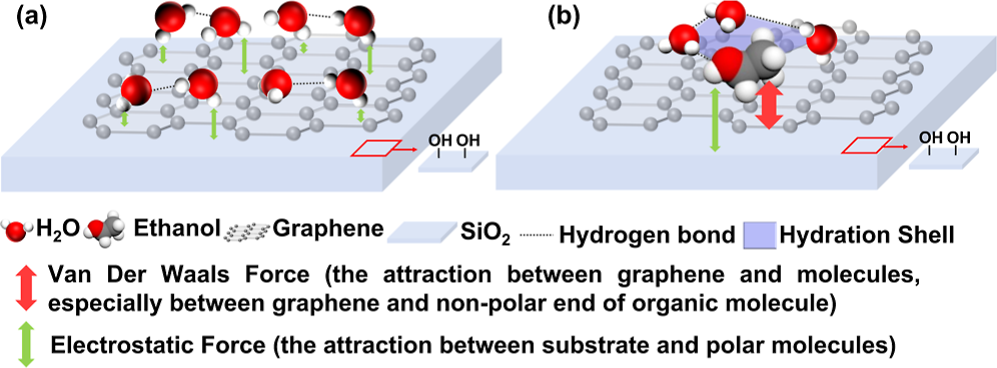
Schematic interfacial diagram of OS-GFET with 10% (*v*/*v*) ethanol aqueous solution: (a) state of water molecules at the graphene–electrolyte interface; (b) state of ethanol molecules at the graphene–electrolyte interface.

The changes in interfacial carriers are influenced by different forces and can be expressed as

5 $$Q_{\left(\Delta V\right)}={-Q}_{\left(EF\right)}-Q_{\left(VdWF\right)}+Q_{\left(HS\right)}$$

Here, *Q*_(EF)_ represents charge accumulation induced by the orientation of organic and water molecules due to electrostatic interactions with the substrate. Equation 5 shows that the van der Waals force, *Q*_(VdWF)_, which corresponds with the adsorption-driven orientation of molecules on graphene, further decreases their distance and amplifies their effect. In addition, the hydration shell’s shielding effect, *Q*_(HS)_, reduces the charge accumulation between interfacial water molecules and organic molecules. Therefore, it decreases the overall charge accumulation and mitigates negative hysteresis. The detailed mechanisms of interfacial evolution are discussed in the Supporting Information (Interfacial Evolution with Hysteresis Formation).

In our experiments measuring organic solvents at 10% (*v*/*v*), we observed negative hysteresis in the OS-GFET measurements (Figure 2). In addition, similar to PS-GFETs, the hysteresis window (HW) of OS-GFETs is inversely proportional to solvent polarity. As previously mentioned, nonpolar domains primarily influence *Q*_(VdWF)_, following the trend: DMSO < ethanol < IPA. In contrast, the other two polarity-driven factors, hydration effects (*Q*_(HS)_) and electrostatic forces (*Q*_(EF)_), follow the opposite trend: DMSO > ethanol > IPA. Notably, *Q*_(VdWF)_ plays a dominant role at the solution/graphene–substrate interface. Consequently, charge accumulation remains negative and |*Q*_(Δ*V*)_| follows the order DMSO < ethanol < IPA.

Furthermore, these effective negative charges induced in supported graphene, e.g., OS-GFETs, increase with rising solvent concentrations and have a near-linear response (Figure 3). This behavior primarily arises from molecular accumulation at the interface. Specifically, as the organic solvent concentration increases, adsorption-induced charge (*Q*_(VdWF)_) and electrostatically driven orientation effects (*Q*_(EF)_) also increase. Meanwhile, as the level of hydration interference (*Q*_(HS)_) is reduced by surface electrostatic interactions, organic molecules accumulate more easily. As a result, overall charge accumulation in graphene is enhanced, further influencing hysteresis behavior.

We also experimentally demonstrated that the sensitivity of OS-GFETs increases nearly linearly as solvent polarity decreases (Figure 4). This behavior arises because polar functional groups primarily influence *Q*_(HS)_ and *Q*_(EF)_, while nonpolar domains exert a stronger impact via *Q*_(VdWF)_. This supports the hypothesis that interfacial changes are predominantly governed by nonpolar interactions. Collectively, these factors regulate interfacial charge dynamics and influence sensor performance.

In brief, the sensing performance of the OS-GFET is governed by a combination of van der Waals and electrostatic interactions. Electrostatic interactions between molecules and the OS-GFET surface enhance van der Waals forces, increasing hysteresis sensitivity and improving molecular differentiation. Notably, within this mechanism, graphene acts synergistically with interfacial van der Waals interactions to modulate carrier transport, resulting in an enhanced sensing performance. van der Waals interactions are primarily mediated through graphene’s delocalized π-electron system, which interacts directly with adsorbed molecules. Understanding these mechanisms is crucial for optimizing GFET-based sensors for applications in aqueous solutions.

### Further Exploration of Substrate Wetting Properties on Sensitivity

Surface wetting behavior is influenced by the electrostatic attraction between water molecules and the interface.^58^ When the substrate surface transitions from hydrophilic to hydrophobic, the electrostatic attraction to polar molecules weakens, leading to an enhanced contact angle. To further investigate the proposed electrostatic attraction-dominated sensing behavior of OS-GFETs, we introduced PMMA and PTFE layers between the SiO_2_ substrate and the graphene layer to modify interface characteristics, as shown in Figure 7a. Experimental results indicate that the contact angles with ethanol for SiO_2_, SiO_2_/PMMA, and SiO_2_/PTFE interfaces are 49°,^59^ 68°,^60^ and 116°^61^, respectively, demonstrating the modification of surface wettability. These changes in wettability are attributed to differences in the chemical composition at the interface. For instance, bare SiO_2_ surfaces contain −OH groups capable of interacting with polar molecules via dipole–dipole interactions and hydrogen bonding, thereby enhancing electrostatic attraction. In contrast, PMMA and PTFE introduce surface chemistries that reduce such interactions: PMMA presents weakly polar ester groups (−COOCH_3_), while PTFE exhibits nonpolar −CF_2_– chains. These modifications lead to a decrease in electrostatic attraction, which is expected to weaken interfacial molecular adsorption.

**Figure 7:**
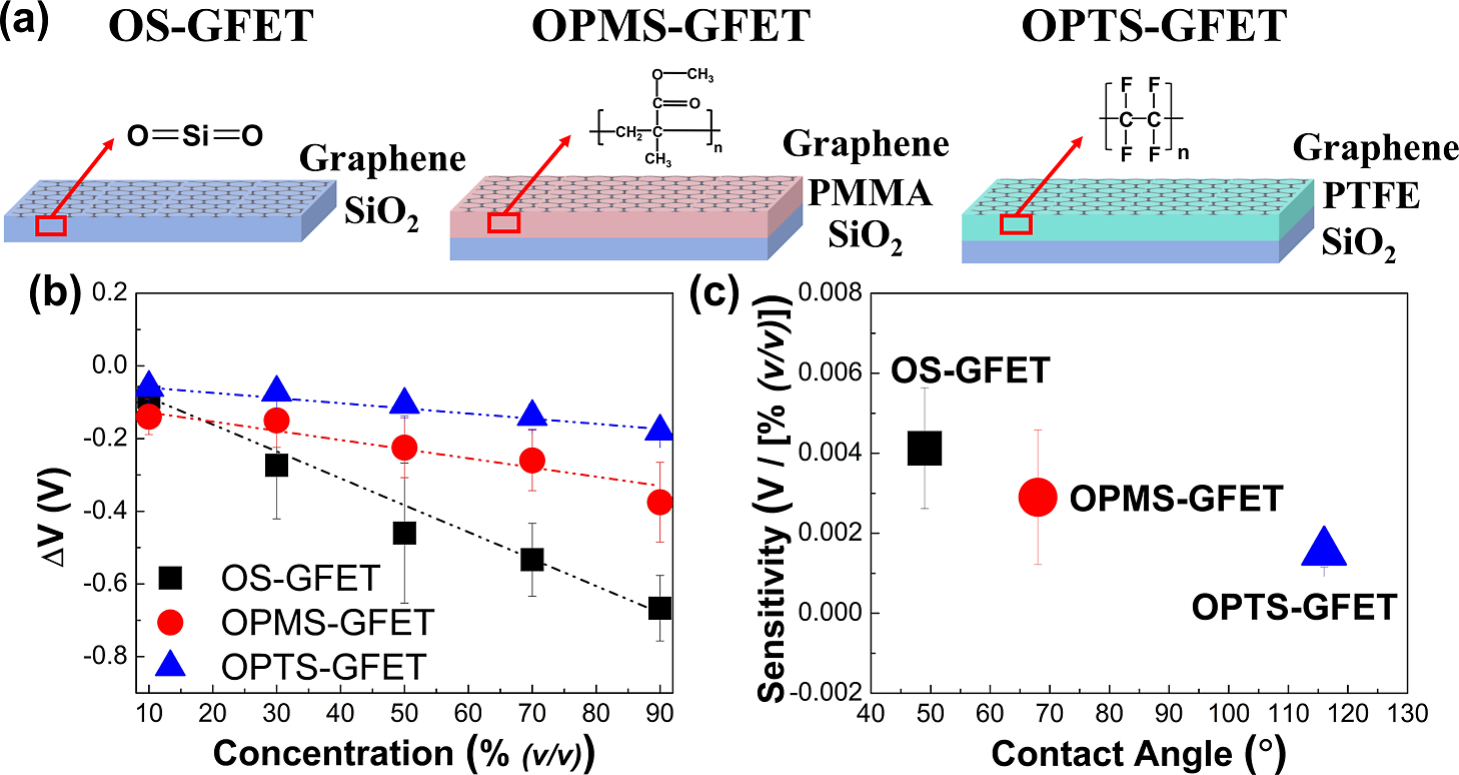
Comparison of the structure and experimental results of OS-GFET, OPMS-GFET, and OPTS-GFET is as follows: (a) the different interfacial layer structure. The red arrows indicate the interfacial molecular structures. (b) Relationship between the ethanol aqueous solutions concentration and related Δ*V* of three different devices. The dotted line represents the fitting line. (c) Relationship between the contact angle and the device sensitivity.

The hysteresis characteristics of OS-GFET, OPMS-GFET, and OPTS-GFET were measured under ethanol aqueous solutions with varying volume concentrations (10%, 30%, 50%, 70%, and 90%), as shown in Figure 7b. As the ethanol concentration increased, the Δ*V* of both OPMS-GFET and OPTS-GFET exhibited a good linear sensing characteristic. Figure 7c illustrates the relationship between the contact angle and device sensitivity. Compared to OS-GFET, both OPMS-GFET and OPTS-GFET show reduced sensitivities. This is primarily due to the reduced interfacial electrostatic attraction to organic molecules. These findings demonstrate that substrate wettability can effectively modulate the sensitivity of aqueous organic solution sensors and support the proposed sensing mechanism of supported-graphene FETs. It should be noted that the substrate-induced doping effect influences the electronic properties; however, the minimal response variation in highly polar solutions (e.g., the response to 10% ethanol in water, as shown in Figure 7b) suggests that interfacial molecular adsorption, rather than doping, governs the sensing behavior. Additionally, the sheet resistance values of OS-GFET, OPMS-GFET, and OPTS-GFET are 417 Ω/□, 450 Ω/□, and 673 Ω/□, respectively. This approach improves transport properties without affecting the electron mobility of graphene.

## Conclusion

This study investigates the sensing capabilities of graphene in organic solvents with varying polarities and different concentrations. In this work, the impact of supporting substrates on graphene-based sensors is emphasized. By examining the electrical responses of partially suspended and substrate-supported graphene, including PS-GFET, OS-GFET, OPMS-GFET, and OPTS-GFET, we reveal key interfacial interactions that dictate the sensing behaviors.

Our findings indicate that OS-GFET exhibits higher sensitivity, greater stability, and a broader linear response range than does PS-GFET. These differences arise from the supporting substrate influence, which enhances van der Waals and electrostatic interactions at the graphene–organic molecule interface. In the OS-GFET, electrostatic attraction drives polar molecules closer to the surface. This strengthens van der Waals interactions and amplifies the hysteresis sensitivity of GFET sensors. This mechanism also enables improved molecular differentiation, particularly for highly polar solvents. In contrast, the hysteresis in PS-GFET originates from the competition between 2D-HBNS and organic hydration clusters. The correlation between the hysteresis sensitivity and interfacial hydration energy highlights the potential of partially suspended graphene for detecting molecules with distinct hydration properties. This mechanism could be extended to applications such as environmental monitoring, where distinguishing molecules based on hydration energy is crucial. Moreover, modifying the wetting properties of the substrate significantly modulates the sensor performance while preserving the intrinsic electrical properties of graphene. These findings underscore the importance of interfacial charge modulation in graphene-based sensing and suggest that tailoring surface properties offers a practical strategy for optimizing molecular recognition. For additional context, a detailed comparison of our findings with previous studies is presented in Supporting Information Table S1.

In conclusion, this work provides insights into the fundamental sensing mechanisms of GFETs and highlights their potential for detecting a wide range of organic compounds. Further validation in diverse applications will be essential for fully realizing these strategies in next-generation sensing technologies.

## Supplementary Material



## Notes

Additional discussions such as electrical measurement range selection, comparative analysis of sensitivity enhancement strategies for molecular sensing, and interfacial evolution of PS-GFET and OS-GFET with hysteresis formation (PDF)

## Abbreviations

PS-GFET: the partially suspended graphene field-effect transistor
OS-GFET: oxide-supported graphene field-effect transistor
OPMS-GFET: oxide-PMMA supported graphene field-effect transistor
OPTS-GFET: oxide-PTFE supported graphene field-effect transistor
HW: hysteresis window
2D-HBNS: two-dimensional hydrogen bond network structure
