# Supplementary material for: An Elucidation of Substrate Effects in Graphene-Based Sensing Characteristics—Interfaces between Organic Solvent and Graphene
Source: ACS Sens. 2025 Jun 17;10(9):6634–44. doi: 10.1021/acssensors.5c01138 (PMC12481550; doi:10.1021/acssensors.5c01138)
Supplement: Supplementary file 1 [file se5c01138_si_001.pdf]

SUPPORTING INFORMATION

An Elucidation of Substrate Effects in Graphene-Based Sensing Characteristics – Interfaces between Organic Solvent and Graphene

Yu-Xuan Lu[1], Guan-Ying Chen[2], Fang-Min Lin[1], Ming-Hsiu Tsai [1], Chih-Ting Lin[*1,2]

1: Graduate Institute of Electronics Engineering, National Taiwan University, Taipei 106319, Taiwan,

2: Graduate Institute of Biomedical Electronics and Bioinformatics, National Taiwan University, Taipei 106319, Taiwan

Corresponding Author: timlin@ntu.edu.tw

1. ELECTRICAL MEASUREMENT RANGE SELECTION

To ensure measurement stability, we restricted the gate voltage sweep to within ±1 V. Due to the fact that the water electrolysis typically occurs around 1.23 V, applying voltages beyond this threshold may trigger reactions such as electrochemical response at the graphene–electrolyte interface. These reactions may alter the interfacial environment by generating gas bubbles, ions, and electrons. Such phenomena would complicate the sensing performance.

We performed comparative experiments with gate voltage sweeps up to ±2 V in ethanol solutions of different concentrations (10%-90%) for both PF-GFET and OS-GFET devices. As shown in Supplementary Figure S1, the transport behavior exhibited no additional peaks behavior. We hypothesize that applying higher temperatures or higher gate voltages would intensify electrochemical phenomena. This justification supports the rationality of our voltage range selection.

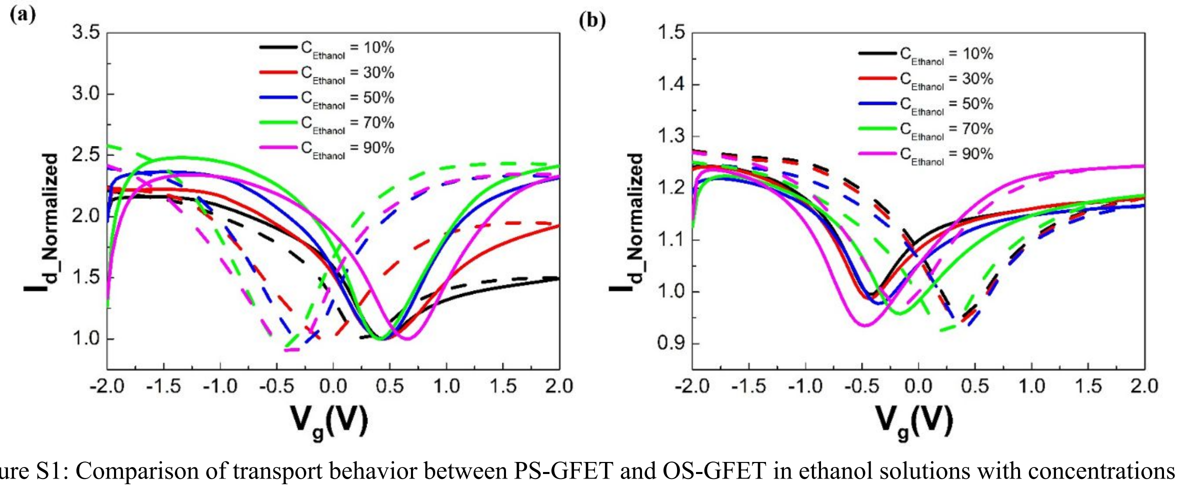

Figure S1: Comparison of transport behavior between PS-GFET and OS-GFET in ethanol solutions with concentrations ranging from 10% to 90% (v/v), measured under gate voltage sweeping from –2 V to +2 V and back to –2 V. Solid lines represent forward scans; dotted lines represent backward scans. (a) PS-GFET; (b) OS-GFET.

2. WATER MOLECULE CONFIGURATION AT PARTIALLY SUSPENDED GRAPHENE INTERFACE

Suspended graphene exhibits hydrophobic characteristics.[1] At hydrophobic interfaces, the establishment of strong interactions between the interface and water molecules is challenging. As a result, water molecules at hydro-phobic interfaces are more likely to dissociate, generating hydroxide (OH^-) and hydrogen (H^+) ions.[2] These dissociated ions form a two-dimensional hydrogen bond network structure (2D-HBNS) with surrounding water molecules. This structure inhibits the recombination of ions. Addition-ally, there are notable differences in the diffusion rates of two hydrated ions in aqueous environments. The diffusion speed of hydroxide ions (OH^-) is significantly slower than the speed of hydrated hydrogen ions (H_3O^+).[3-6]

The layered distribution of ions affects the electrical transport properties of the graphene channel. This difference becomes more pronounced during the dynamic modulation process. When the gate voltage (V_g) is negative, the electric field drives more OH^- ions to accumulate in the first water layer near the graphene interface. Meanwhile, H^+ ions tend to reside in the outer second water layer. As Vg gradually increases from negative to positive voltage, H^+ ions migrate towards the first layer gradually. At this process, H^+ ions combine with OH^- ions, and they form dangling water molecules.[7] These dangling water molecules represent

a collection of free-state water molecules that reorient much more quickly than typical water molecules, often orienting towards the surface.[8,9] When the gate voltage gradually decreases at backward sweep, these dangling water molecules rapidly reform the stable 2D-HBNS structure. The changes of the 2D-HBNS structure during the forward and backward sweeping processes lead to differences in the modulation of graphene band structure. This alteration in electrical performance corresponds with the hysteresis effect caused by water.[5, 6]

When the concentration of aqueous organic solution is 10% *v/v*, water molecules remain the primary component and they play a dominant role at the interface. Due to the presence of a small amount of organic molecules, some water molecules form hydration shell around these molecules. As a result, the energy barrier for the dissociation of these oriented water molecules increases. This requires the water molecules to desorb from the organic hydration shell before dissociation can occur. As a result, the generation of hydroxide (OH^-) and hydrogen (H^+) ions at the interface is inhibited. This process directly affects the formation of the interfacial two-dimensional hydrogen bond network structure (2D-HBNS). As a result, there is a noticeable decrease in the 2D-HBNS structure during electrical modulation, especially at the backward sweep.

As the gate voltage transitions to positive during the forward voltage sweep, the interfacial water molecules point towards the graphene surface and form hydration bond with silanol groups. When the gate voltage transitions from positive to zero in the backward sweep, it is difficult for these oriented water molecules to reform 2D-HBNS. This causes a negative shift in the charge neutrality point, which subsequently leads to a negative hysteresis effect. Interfacial organic molecules are influenced by SiO_2 substrate, graphene and water molecules.

3. INTERFACIAL EVOLUTION WITH HYSTERESIS FORMATION

Interface changes are a critical factor driving the distinct hysteresis behaviors observed. In this section, we will delve into the mechanisms underlying this phenomenon by examining the interface changes when PS-GFET and OS-GFET are in contact with a 50% (*v/v)* ethanol aqueous solution.

Figure S2 illustrates the evolution of the interface during the gate voltage sweeping process in an ethanol aqueous solution for PS-GFET. At the initial stage of the positive voltage sweep, the gate electrode applies a negative voltage to the solution. This change in electric potential draws OH^- ions closer to the graphene surface while H^+ ions move away, thereby strengthening the two-dimensional hydrogen bond network structure (2D-HBNS) at the graphene-electrolyte interface. For ethanol, a polar protic solvent, its oxygen atom orients towards the graphene surface, as shown in Figure S2(a). Hydrogen bonds form between organic molecules and water molecules, leading to the gradual formation of hydration clusters at the interface. The hydrogen bonds formed between organic molecules and water molecules are indicated by blue dashed lines in the figure. For simplicity, surrounding water molecules near the 2D-HBNS and hydration clusters are omitted in the figure. At this stage, the interface configuration gives graphene a p-type doping characteristic. As the gate voltage increases towards positive potential, H^+ ions are attracted to the graphene surface, resulting in the gradual breakdown of the 2D-HBNS, as depicted in Figure S2(b). This disruption significantly weakens the hydrogen bonding interactions, leading to an increase in the number of water molecules at the interface. Some water molecules exist as dangling water, while others form hydration clusters with ethanol molecules at the interface. Simultaneously, the ethanol molecules reorient in response to the electric field changes, a process occurring on the picosecond scale and much faster than the response time of the measurement system, thus having negligible impact on the transient electrical behavior. During this phase, the electron doping of the graphene surface increases noticeably. In the reverse voltage sweeping process, as the positive potential gradually decreases, the concentration of ions (H_3O^+ and OH^-) at the interface increases, which strengthens the 2D-HBNS structure and decreases the corresponding electron doping, as shown in Figure S2(c). As the gate voltage shifts to negative potential, the hydration structure of the 2D-HBNS with the organic molecules is further enhanced, as illustrated in Figure S2(d). This change in interface configuration increases the negative charge at the interface, leading to the observed hysteresis effect. Figure S2(e) provides a detailed view of the fermi level changes in the graphene channel at different gate voltages, corresponding to the analyses in Figure S2(a) – (d). As the gate voltage and interface configuration vary, the electron density and carrier concentration in the graphene also change. For PS-GFET, the variation of OH^- ions at the interface plays a crucial role in the doping of the channel. The redistribution of OH^- ions directly affects the carrier concentration in the graphene channel, thereby modulating its electronic properties.

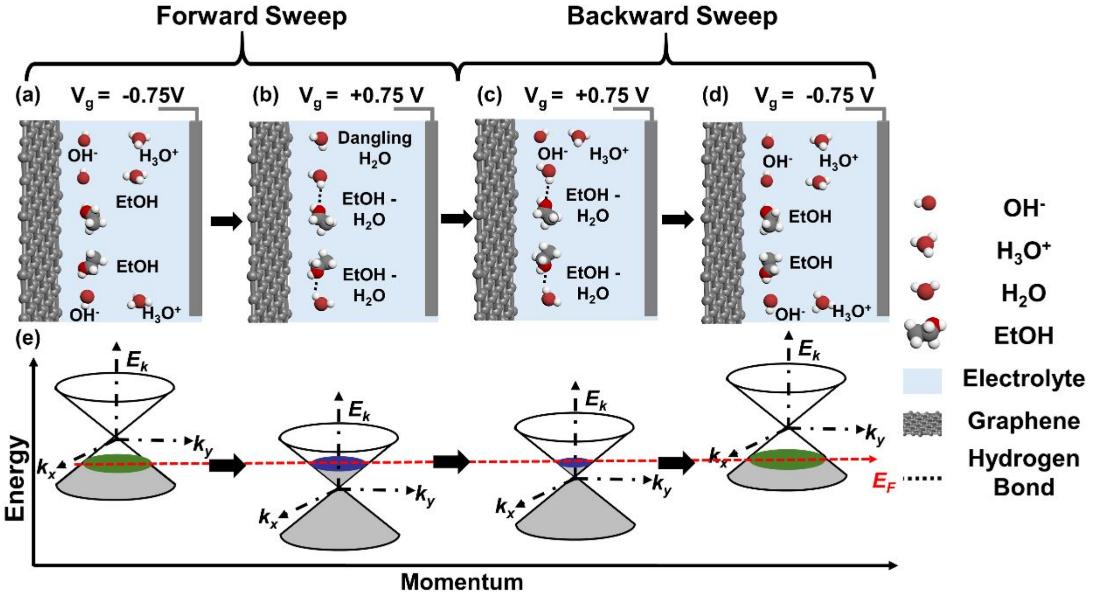

Figure S2: Schematic of interfacial evolution of PS-GFET with ethanol (EtOH) aqueous solution (50% (*v/v*)) electrolyte solution with different applied gate voltages. (a) $V_g = -0.75$ V at the forward sweep path. (b) $V_g = +0.75$ V at the forward sweep path. (c) $V_g = +0.75$ V at the backward sweep path. (d) $V_g = -0.75$ V at the backward sweep path. (f) Fermi level variation of graphene at modulated with corresponding electrical field.

In contrast to the PS-GFET, the electrical results of the OS-GFET exhibit distinct behavior. Figure S3 presents a schematic representation of the interfacial evolution of the OS-GFET in an ethanol aqueous solution during the gate-voltage sweeping process. At the initial stage of the positive voltage sweep, the applied electric potential causes the oxygen atoms in the ethanol molecules to orient towards the surface, which results in the dipole moment of the ethanol molecules pointing away from the graphene interface, as illustrated in Figure S3(a). Since graphene remains hydrophobic at the interface, both OH^- and H^+ ions exist on the graphene surface. During this phase, water molecules can form a two-dimensional hydrogen bond network structure (2D-HBNS) with the ions or hydrate with the organic molecules. Hence, graphene displays p-type doping characteristics due to the interface configuration. As the gate voltage increases to positive values, as shown in Figure S3(b), the hydrogen atoms in the ethanol molecules are increasingly oriented towards the surface, causing the dipole moment of the ethanol molecules to shift towards the graphene interface. The negative potential from the SiO_2 substrate causes the organic molecules in the first layer of water closer to the graphene surface. Additionally, the Van der Waals attraction from the graphene strengthens for these organic molecules as their distance decreases, as indicated by the red arrows in the figure. Water molecules also orient towards the surface in response to the electric potential, exhibiting collective dipole behavior. The water molecules pointing toward the interface have electrostatic force with the substrate, as illustrated by the blue dashed lines in the figure. At this stage, electron doping on the graphene surface significantly increases.

When the gate voltage reaches +1 V, more polar molecules accumulate at the interface under the influence of the electric potential. As the gate voltage is back to +0.75 V, the number of water molecules at the interface decreases with the lowering of the potential. However, ethanol molecules become more difficult to detach from the surface due to the Van der Waals forces from the graphene, as depicted in Figure S3(c). The increase in organic molecules and the orientation of water towards the substrate together lead to an increased electron doping in the graphene channel compared to the forward sweep to $V_g = +0.75$ V. As the gate voltage further decreases to -0.75 V, 2D-HBNS and organic hydration shell reform at the interface, as shown in Figure S3(d). Due to the Van der Waals force between graphene and organic molecules, organic molecules are closer to the graphene surface than water molecules. At this stage, compared with the same gate voltage at the forward sweeping process. The formation of the 2D become more difficult. Hence, the hole doping level is lower compared to the positive sweep condition. Figure S3(e) details the energy band changes in the graphene channel at different gate voltages, corresponding to the analyses in Figure S3(a) – (d). Unlike the significant role of surface OH^- variation in channel doping for PS-GFET during electrical modulation, the increase in organic molecules and the collective orientation of water molecules at the OS-GFET interface serve as the main source of hysteresis performance. The increase in the number of organic molecules and their interaction with the graphene interface enhances the interfacial capacitive gating effect, thereby influencing the doping behavior of the graphene channel.

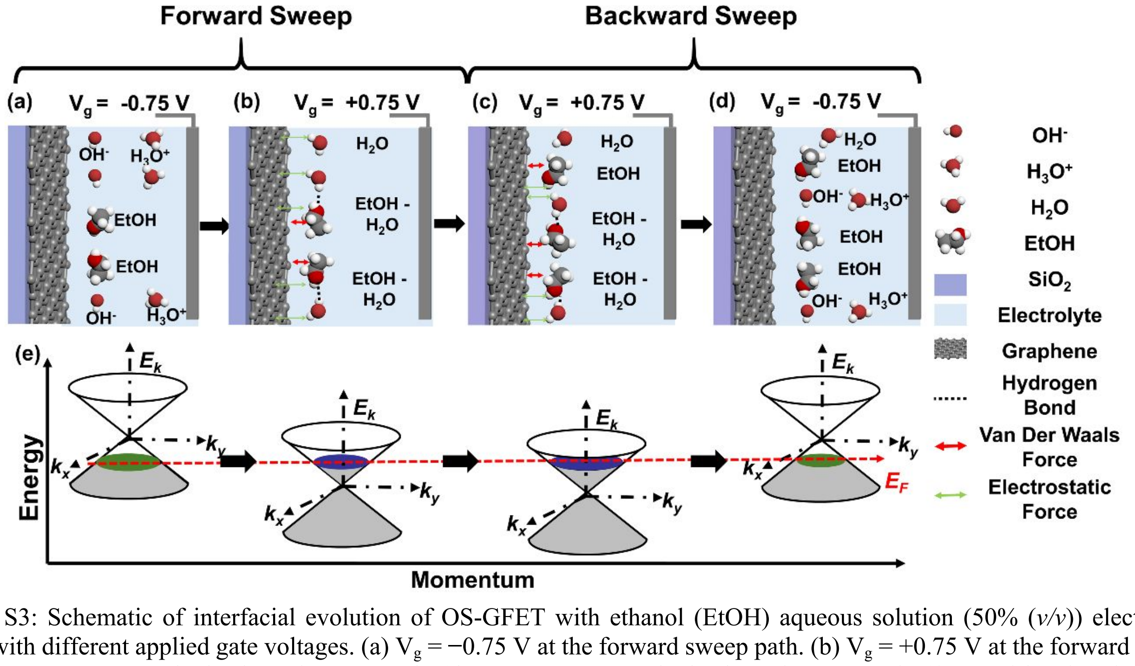

Figure S3: Schematic of interfacial evolution of OS-GFET with ethanol (EtOH) aqueous solution (50% (*v/v*)) electrolyte solution with different applied gate voltages. (a) $V_g = -0.75$ V at the forward sweep path. (b) $V_g = +0.75$ V at the forward sweep path. (c) $V_g = +0.75$ V at the backward sweep path. (d) $V_g = -0.75$ V at the backward sweep path. (f) Fermi level variation of graphene at modulated with corresponding electrical field.

4. Comparison of Improvement Strategies for Graphene-Based Sensors in Small Organic Molecule Detection

Table S1 provides a comparative overview of representative sensitivity enhancement strategies for graphene-based sensors targeting small organic molecules. These methods are categorized by sensing mechanisms—electrochemical and chemiresistive. For each strategy, the table outlines the advantages, limitations, and typical target analytes, offering a comprehensive overview of their applicability to gas-phase and liquid-phase organic molecule detection.

As shown in the table, commonly employed techniques include metal nanoparticle decoration, metal oxide integration, conductive polymer incorporation, atomic doping, functional group modification, non-covalent molecular functionalization,

ionic liquid (IL) modification, and porous structure engineering. Most of these enhance sensitivity through surface functionalization. However, such approaches often suffer from poor long-term stability in liquid environments, non-uniform doping, and degradation of graphene's intrinsic electronic properties due to direct interfacial modification.

In contrast, our method leverages physisorption-driven mechanism, where a hydrophilic substrate promotes electrostatic interactions between dipolar organic molecules and the graphene interface. This non-covalent strategy achieved solely by substituting the supporting substrate, preserves the pristine electronic characteristics of graphene. As a result, sensing performance can be modulated through a simple structure design, offering excellent long-term stability and reproducibility in organic environments.

Although the current level of sensitivity enhancement is moderate, the focus of our work lies in mechanistic innovation. We introduce an electrostatics-based strategy that offers a new direction for improving sensor performance. Such a direction remains largely underexplored, particularly for detecting small polar organic molecules in solution.

Table S1: Summary of Improvement Strategies for Graphene-Based Sensors in Small Organic Molecule Detection

**Sensing Mechanism**	**Improvement methods**	**Mechanism Principle**	**Advantage**	**Limitation**	**Representative Analyte**	**Reference**
Electrochemical	Metal nanoparticles modification (e.g., Pt, Au, Pd)	Provide high catalytic activity and electron transfer channels	– Improved sensitivity<br>– Suitable for low-concentration detection	– Limited long-term stability<br>– High cost	Glucose, Paracetamol	[10, 11]
	Metal oxides modification (e.g., Cu_2O, SnO_2)	High surface activity, facilitates redox reactions	– Low cost<br>– Enhanced sensitivity and selectivity	– Low conductivity<br>– Limited stability	Dopamine, Glucose	[12, 13]
	Conductive polymers modification (e.g., polyaniline, polypyrrole)	Enhance conductivity and provide active sites	– High selectivity<br>– Easy to functionalize	– Poor stability<br>– Susceptible to degradation	Adenine, Adenine, Guanine	[14, 15]
	Atomic doping (e.g., P, N)	Provides active sites ,improves interfacial charge transfer and redox reactivity	– Boosts electrocatalytic activity and sensitivity	– Affects graphene conductivity<br>– Non-uniform doping affects reproducibility	Acetaminophen, Dopamine	[16, 17]
	Functional group modification (e.g., –COOH, $–NH_2$)	Provides active sites ,improves interfacial charge transfer and redox reactivity	– Boosts electrocatalytic activity and sensitivity	– Affects graphene conductivity<br>– Non-uniform functionalization affects reproducibility	Dopamine	[18]
	Non-covalent molecular functionalization (e.g., π–π stacking)	Preserve conjugated structure and conductivity of graphene	– Non-destructive<br>– Simple operation	– Poor functionalization stability	Glucose	[19]

	Ionic liquid (IL) modification	Enhance conductivity and increase active sites	– Improved conductivity<br>– More active sites	– Limited long-term stability	Catechol, Hydroquinone, Caffeic acid	[20, 21]
	Porous structure (3D rGO)	Improves electrolyte–electrode contact area for enhanced redox reactions	– Boosts sensitivity and signal stability<br>– Promotes efficient analyte diffusion in liquids	– Susceptible to biofouling and ion trapping<br>– Irregular porosity affects reproducibility<br>– May collapse under long-term cycling	Glucose, Dopamine	[22, 23]
Chemiresistive	Metal nanoparticles (e.g., Pt, Au, Pd)	Enhance adsorption of polar molecules and amplify resistance change	– High surface energy and catalytic properties enable rapid detection	–Unstable modification layer lowers reproducibility and stability	Acetic acid, Acetone, Ethanol	[24, 25]
	Metal oxides (e.g., ZnO, SnO_2)	Enhance molecular adsorption and electron transfer ability	– Good response to gases	– Poor conductivity and stability in water– risk of electrical drift in liquid	Ethanol, Acetone	[26, 27]
	Conductive polymers (e.g., polyaniline, polypyrrole)	Enhance conductivity and reaction capability	– Good stability, potential for flexible substrates	– Humidity-sensitive polymer swelling must be controlled in liquid	Acetone, Ethanol, Methanol	[28, 29]
	Atomic doping (e.g., N, Bi)	Enhance target molecule adsorption and charge modulation	– Improved selectivity and sensitivity	– Affects graphene conductivity<br>– Non-uniform doping affects reproducibility	Formaldehyde, Ethanol, Methanol	[30, 31]
	Functional group modification (e.g., C=O)	Enhance target molecule adsorption and charge modulation	– Improved selectivity and sensitivity	– Affects graphene conductivity<br>– Non-uniform doping affects reproducibility	Methanol, Ethanol	[32]
	Porous structure	Increases effective area for charge transfer across adsorbed molecules	– Boosts sensitivity and signal stability<br>– Increases gas response area	– Sensitive to humidity and environmental drift	Ethanol, Acetone	[33]

Physisorption-induced charge modulation	Interfacial electrostatic effect	Enhance molecular adsorption by electrostatic effect through substrate-induced effects	– Improved sensitivity<br>– Simple structure<br>– High stability<br>– Preserves intrinsic properties	– Structural similarity among molecules limits selectivity	Ethanol, Isopropanol Alcohol, Dimethyl sulfoxide	This work

REFERENCES

(1) Fan, X.; Yang, S.; Huang, C.; Lu, Y.; Dai, P. Preparation and enhanced acetone-sensing properties of ZIF-8-derived Co_3O_4@ZnO microspheres. Chemosensors 2023, 11, 376.

(2) Kudin, K. N.; Ruoff, R. C. Why are water hydrophobic interfaces charged? *J. Am. Chem. Soc*. **2008**, 130, 3915-3919.

(3) Chen, M.; Zheng, L.; Santra, B.; Ko, H. Y.; DiStasio, R. A., Jr.; Klein, M. L.; Car, R.; Wu, X. Hydroxide diffuses slower than hydronium in water because its solvated structure inhibits correlated proton transfer. *Nat. Chem*. **2018**, 10, 413-419.

(4) Marx, M. E. T. C. Structure and dynamics of OH^-(aq). *Acc. Chem. Res.* 2006, 39, 151-158.

(5) Tsai, M. H.; Lu, Y. X.; Lin, C. Y.; Lin, C. H.; Wang, C. C.; Chu, C. M.; Woon, W. Y.; Lin, C. T. The first-water-layer evolution at the graphene/water interface under different electro-modulated hydrophilic conditions observed by suspended/supported field-effect-device architectures. *ACS Appl. Mater. Interfaces* **2023**, 15, 17019-17028.

(6) Lu, Y. X., Tsai, M. H., Lin, C. Y., Woon, W. Y., Lin, C. T. Nanoscopic Supercapacitance Elucidations of the Graphene-Ionic Interface with Suspended/Supported Graphene in Different Ionic Solutions. *ACS Appl. Mater. Interfaces* **2025**, 17, 5419-5429.

(7) Stirnemann, G.; Castrillon, S. R.; Hynes, J. T.; Rossky, P. J.; Debenedetti, P. G.; Laage, D. Non-monotonic dependence of water reorientation dynamics on surface hydrophilicity: competing effects of the hydration structure and hydrogen-bond strength. *Phys. Chem. Chem. Phys.* **2011**, 13, 19911-19917.

(8) Zhang, Y.; Stirnemann, G.; Hynes, J. T.; Laage, D. Water dynamics at electrified graphene interfaces: a jump model perspective. *Phys. Chem. Chem. Phys.* **2020**, 22, 10581-10591.

(9) Zhang, Y.; de Aguiar, H. B.; Hynes, J. T.; Laage, D. Water structure, dynamics, and sum-frequency generation spectra at electrified graphene interfaces. *J. Phys. Chem. Lett.* **2020**, 11, 624-631.

(10) Aslan, S.; Anik, M. Microbial glucose biosensors based on glassy carbon paste electrodes modified with Gluconobacter oxydans and graphene oxide or graphene–platinum hybrid nanoparticles. *Microchim. Acta* **2016**, 183, 73–81

(11) Ye, Y.; Ding, S.; Ye, Y.; Xu, H.; Cao, X.; Liu, S.; Sun, H. Enzyme-based sensing of glucose using a glassy carbon electrode modified with a one-pot synthesized nanocomposite consisting of chitosan, reduced graphene oxide and gold nanoparticles. *Microchim. Acta* **2015**, 182, 1783–1789.

(12) Rao, D.; Zhang, X.; Sheng, Q.; Zheng, J. Highly improved sensing of dopamine by using glassy carbon electrode modified with MnO_2, graphene oxide, carbon nanotubes and gold nanoparticles*. Microchim. Acta* **2016**, 183, 2597–2604

(13) Ma, H.-F.; Chen, T.-T.; Luo, Y.; Kong, F.-Y.; Fan, D.-H.; Fang, H.-L.; Wang, W. Electrochemical determination of dopamine using octahedral SnO_2 nanocrystals bound to reduced graphene oxide nanosheets. *Microchim. Acta* **2015**, 182, 2001–2007.

(14) Sharma, V.; Hynek, D.; Trnkova, L.; Hemzal, D.; Marik, M.; Kizek, R.; Hubalek, J. Electrochemical determination of adenine using a glassy carbon electrode modified with graphene oxide and polyaniline. *Microchim. Acta* **2016**, 183, 1299–1306

(15) Gao, Y. S.; Xu, J. K.; Lu, L. M.; Wu, L. P.; Zhang, K. X.; Nie, T.; Zhu, X. F.; Wu, Y. Overoxidized polypyrrole/graphene nanocomposite with good electrochemical performance as novel electrode material for the detection of adenine and guanine. *Biosens. Bioelectron*. **2014**, 62, 261–267.

(16) Zhang, X.; Wang, K. P.; Zhang, L. N.; Zhang, Y. C.; Shen, L. Phosphorus-doped graphene-based electrochemical sensor for sensitive detection of acetaminophen. *Anal. Chim. Acta* **2018**, 1036, 26–32.

(17) Feng, X.; Zhang, Y.; Zhou, J.; Li, Y.; Chen, S.; Zhang, L.; Yan, X. Three-dimensional nitrogen-doped graphene as an ultrasensitive electrochemical sensor for the detection of dopamine. *Nanoscale* **2015**, 7, 2427–2432.

(18) Rahman, M. M.; Liu, D.; Lopa, N. S.; Baek, J. B.; Nam, C. H.; Lee, J. J. Effect of the carboxyl functional group at the edges of graphene on the signal sensitivity of dopamine detection. *J. Electroanal. Chem.* **2021**, 898, 115628.

(19) Chia, J. S. Y.; Tan, M. T.; Khiew, P. S.; Chin, J. K.; Siong, C. W. A bio-electrochemical sensing platform for glucose based on irreversible, non-covalent π–π functionalization of graphene produced via a novel, green synthesis method. *Sens. Actuators, B* **2015**, 210, 558–565.

(20) Wang, C.; Chen, Y.; Zhuo, K.; Wang, J. Simultaneous reduction and surface functionalization of graphene oxide via an ionic liquid for electrochemical sensors. *Chem. Commun.* **2013**, 49, 3336–3338.

(21) Valentini, F.; Roscioli, D.; Carbone, M.; Conte, V.; Floris, B.; Bauer, E. M.; Ditaranto, N.; Sabbatini, L.; Caponetti, E.; Chillura-Martino, D. Graphene and ionic liquids new gel paste electrodes for caffeic acid quantification. *Sens. Actuators, B* **2015**, 212, 248–255.

(22) Zhao, Y.; Bo, X.; Guo, L. Highly exposed copper oxide supported on three-dimensional porous reduced graphene oxide for non-enzymatic detection of glucose. *Electrochim. Acta* **2015**, 176, 1272–1279.

(23) Yu, B.; Kuang, D.; Liu, S.; Liu, C.; Zhang, T. Template-assisted self-assembly method to prepare three-dimensional reduced graphene oxide for dopamine sensing. *Sens. Actuators B* **2014**, 205, 120–126.

(24) Gautam, M.; Jayatissa, A. H. Detection of organic vapors by graphene films functionalized with metallic nanoparticles. *J. Appl. Phys.* **2012**, 112, 114324.

(25) Dhall, S.; Kumar, M.; Bhatnagar, M.; Mehta, B. R. Dual gas sensing properties of graphene–Pd/SnO_2 composites for H_2 and ethanol: Role of nanoparticles–graphene interface. *Int. J. Hydrogen Energy* **2018**, 43, 17921–17927.

(26) Liang, S.; Zhu, J.; Ding, J.; Bi, H.; Yao, P.; Han, Q.; et al. Deposition of cocoon-like ZnO on graphene sheets for improving gas-sensing properties to ethanol. *Appl. Surf. Sci.* **2015**, 357, 1593–1600.

(27) Zhang, H.; Cen, Y.; Du, Y.; Ruan, S. Enhanced acetone sensing characteristics of ZnO/graphene composites. *Sensors* **2016**, 16, 1876.

(28) Deb, K.; Debnath, A.; Bera, A.; Sarkar, K.; Debnath, A.; Saha, B. Polyaniline encapsulated graphite: A sensitive system for resistive detection of methanol. *Surf. Interfaces* **2019**, 16, 141–146.

(29) Zamiri, G.; Haseeb, A. S. M. A. Recent trends and developments in graphene/conducting polymer nanocomposites chemiresistive sensors. *Materials* **2020**, 13, 3311.

(30) Zhang, H.; Luo, X.; Lin, X.; Lu, X.; Leng, Y.; Song, H. Density functional theory calculations on the adsorption of formaldehyde and other harmful gases on pure, Ti-doped, or N-doped graphene sheets. *Appl. Surf. Sci.* **2013**, 283, 559–565.

(31) Cai, S. X.; Song, X. Q.; Chi, Z. T.; Fu, Y. Q.; Fang, Z. T.; Geng, S. Y. Y.; et al. Rational design of Bi-doped rGO/Co_3O_4 nanohybrids for ethanol sensing. *Sens. Actuators B* **2021**, 343, 130118.

(32) Rabchinskii, M. K.; Sysoev, V. V.; Varezhnikov, A. S.; Solomatin, M. A.; Struchkov, N. S.; Stolyarova, D. Y.; et al. Toward on-chip multisensor arrays for selective methanol and ethanol detection at room temperature: Capitalizing the graphene carbonylation. *ACS Appl. Mater. Interfaces* **2023**, 15, 28370–28386.

(33) Jang, J. S.; Lee, J.; Koo, W. T.; Kim, D. H.; Cho, H. J.; Shin, H.; Kim, I. D. Pore-size-tuned graphene oxide membrane as a selective molecular sieving layer: Toward ultraselective chemiresistors. *Anal. Chem.* **2020**, 92, 957–965.
